# Assessing the real-world value of hemophilia care: how suitable are patient-centered outcomes for hospital-level comparisons?

**DOI:** 10.1093/intqhc/mzag105

**Published:** 2026-08-04

**Authors:** Diaz M Prameyllawati, Samantha C Gouw, Hester F Lingsma, Marjon H Cnossen, Floor C J I Heubel-Moenen, Frank W G Leebeek, Nick van Es, Johanna G van der Bom, Renske M T ten Ham, Martijn A H Oude Voshaar

**Affiliations:** Department of Public Health, Erasmus MC, University Medical Center Rotterdam, Rotterdam, 3015 GD, The Netherlands; Department of Epidemiology and Health Economics, Julius Center for Health Sciences and Primary Care, University Medical Center Utrecht, Utrecht, 3584 CX, The Netherlands; Department of Pediatric Hematology, Amsterdam UMC location University of Amsterdam, Amsterdam, 1105 AZ, The Netherlands; Department of Clinical Epidemiology, Leiden University Medical Center, Leiden, 2333 ZA, The Netherlands; Department of Public Health, Erasmus MC, University Medical Center Rotterdam, Rotterdam, 3015 GD, The Netherlands; Department of Pediatric Hematology and Oncology, Erasmus MC Sophia Children’s Hospital, University Medical Center Rotterdam, Rotterdam, 3015 GD, The Netherlands; Department of Hematology, Maastricht University Medical Center and Hemophilia Treatment Center Nijmegen-Eindhoven-Maastricht, Maastricht, 6229 HX, The Netherlands; Cardiovascular Research Institute Maastricht (CARIM), Maastricht University, Maastricht, 6211 LK, The Netherlands; Department of Hematology, Erasmus MC, University Medical Center Rotterdam, Rotterdam, 3015 GD, The Netherlands; Department of Vascular Medicine, Amsterdam University Medical Center, Amsterdam, 1105 AZ, The Netherlands; Department of Clinical Epidemiology, Leiden University Medical Center, Leiden, 2333 ZA, The Netherlands; Department of Epidemiology and Health Economics, Julius Center for Health Sciences and Primary Care, University Medical Center Utrecht, Utrecht, 3584 CX, The Netherlands; Department of Public Health, Erasmus MC, University Medical Center Rotterdam, Rotterdam, 3015 GD, The Netherlands

**Keywords:** hemophilia, patient outcome, hospital comparison, real-world data, value-based healthcare

## Abstract

**Background:**

Hospital-level comparisons of patient-centered outcomes play a central role in value-based healthcare. In hemophilia care, several standardized sets of patient-centered outcomes have been developed to foster the implementation of value-based healthcare; however, their suitability for hospital-level comparisons has not yet been assessed. Therefore, we evaluated the suitability of patient-centered outcomes for hospital-level comparisons in hemophilia care.

**Methods:**

We analyzed data from 807 adults with hemophilia enrolled in the 2019 HiN-6 study in the Netherlands. Twelve patient-centered outcomes were assessed. The suitability of these outcomes for hospital comparisons was evaluated using two criteria: (i) sensitivity to case mix, evaluated by Spearman’s rank correlation between case-mix-adjusted and case-mix-unadjusted hospital-level scores and (ii) reliability of between-hospital differences, determined by rankability defined as the proportion of between-hospital variation not attributable to chance. High correlations indicate a minimal case-mix influence, while a high rankability reflects a high degree of reliability.

**Results:**

Most patient-centered outcomes were relatively insensitive to case mix (*ρ* > 0.70). Reliability of hospital differences was generally low (rankability <0.50). Only the annual bleeding rate and annual joint bleeding rate demonstrated moderate reliability with a rankability of 0.55 and 0.58, respectively.

**Conclusion:**

Most patient-centered outcomes assessed were not suitable for comparing hemophilia care at the hospital level because of low reliability. Among the outcomes evaluated, the annual bleeding rate and annual joint bleeding rate appeared relatively more suitable for such comparisons.

## Introduction

Hemophilia is an inherited X-linked bleeding disorder that results from a deficiency or dysfunction of coagulation factor VIII (hemophilia A) or factor IX (hemophilia B) [1]. Hemophilia A and B are rare, affecting 24.6 and 5.0 per 100 000 male newborns, respectively [1, 2]. Over the years, treatment for hemophilia has evolved significantly, from clotting factor replacement to gene therapy [3, 4]. With ongoing advances in hemophilia therapies, increasing costs, and care management, there is growing interest in adopting value-based healthcare (VBHC) practices within this field. The implementation of VBHC enables healthcare systems to optimize patient outcomes and resource utilization while promoting consistent value across hospitals through cross-institutional learning.

Patient-centered outcomes, defined as health outcomes that are meaningful to patients, are central to VBHC [5]. Hemophilia care has increasingly moved toward standardized outcome measurement through coordinated stakeholder efforts [6, 7]. This is exemplified by two major consensus initiatives designed to establish core outcome sets that emphasize patient-centeredness. In 2019, van Balen *et al.* introduced an international standard outcome set intended to optimize care for hemophilia [6]. This core set consists of 10 health outcomes, formulated according to the International Consortium for Health Outcomes Measurements (ICHOM) methodology [6]. In 2024, a consensus-based outcome set for evaluating the quality of hemophilia care was published by Cortesi *et al.* [7]. This set was also developed using the VBHC approach and consists of eight health outcomes and two composite outcomes.

Following the establishment of standardized outcome sets for hemophilia, the ICHOM advocates for the systematic measurement of these outcomes and their consistent comparison across institutions [8]. Such an approach enables the identification of more effective treatment strategies and promotes improvements in overall healthcare outcomes. While several of the patient-centered outcomes in the two standardized outcome sets overlap with measures that are used in clinical trials in hemophilia such as bleeding events and joint health and are well-validated for patient-level analyses [9], their suitability for comparing hospitals—rather than individual patients—has not been comprehensively evaluated.

Hospital-level comparisons are inherently challenging, as case-mix differences and nonrandom hospital selection limit causal inference, while small sample sizes and modest between-hospital variation compromise reliability [10]. Meaningful hospital-level comparisons therefore require careful consideration of these methodological challenges. Before such comparisons can be made, the suitability of patient-centered outcomes must be evaluated to ensure that they are capable of distinguishing true between-hospital differences from random variation and are either unaffected by or appropriately adjusted for case mix. Therefore, the current study aims to evaluate the suitability of patient-centered outcomes for hospital-level comparisons in hemophilia care in the Netherlands.

## Materials and methods

### Data

We utilized data from the Hemophilia in the Netherlands-6 (HiN-6) study, a nationwide study conducted in 2018–9 with the aim of including the entire Dutch hemophilia population (further details about the HiN-6 study are provided in Supplementary Appendix S1) [11]. The study was approved by the Medical Ethics Committee of Leiden University Medical Center in 2018. For the present analysis, inclusion criteria required participants to be adults and to have completed both questionnaire data and given consent for data collection from their medical record. Applying these criteria resulted in the inclusion of 807 adult participants from the HiN-6 study.

### Conceptual framework for assessing outcome measures

We applied the framework for meaningful hospital comparisons developed by Lingsma (Supplementary Appendix Fig. S1) [10]. According to this framework, observed hospital differences can be divided into random variation, variation due to case mix, and unexplained variation. The latter may be residual confounding or registration bias, while the remaining between-center variation may reflect true between-hospital differences in outcome. In this study, we focused on assessing case-mix effects and quantifying the proportion of between-hospital variation not attributable to chance to evaluate the suitability of the selected outcomes for hospital comparisons. A total of 12 case-mix variables and 12 outcome measures were included (Tables 1 and 2), selected according to predefined procedures detailed in Supplementary Appendix S2.

**Table 1:** Selected case-mix variables for evaluation of outcome measures.

Domain	Case-mix variable	Measurement instrument HiN-6 study
Age	Age	Questionnaire item: “What is your age?”
Socioeconomic status	Education	Questionnaire item: “What is the highest level of education you have completed with a diploma?”
	Annual income	Questionnaire item: “What is the gross income of your household?”
Comorbidities	Hypertension	Questionnaire item: “Are you currently being treated by a medical specialist or general practitioner for hypertension?”
	Hypercholesterolemia	Questionnaire item: “Are you currently being treated by a medical specialist or general practitioner for hypercholesterolemia?”
	Diabetes mellitus type 1	Questionnaire item: “Are you currently being treated by a medical specialist or general practitioner for diabetes mellitus type 1?”
	Diabetes mellitus type 2	Questionnaire item: “Are you currently being treated by a medical specialist or general practitioner for diabetes mellitus type 2?”
	COPD	Questionnaire item: “Are you currently being treated by a medical specialist or general practitioner for COPD?”
	Hepatitis C	Questionnaire item: “Are you currently being treated by a medical specialist or general practitioner for hepatitis C?”
Severity of hemophilia	Severity	Questionnaire item: “What is the severity of your hemophilia?”
Psychological well-being	Psychological complaints	Questionnaire item: “Are you currently being treated by a medical specialist or general practitioner for psychological complaints (depression, anxiety disorder, psychosis, etc.)”
Inhibitor status	Inhibitor	Questionnaire item: “Have you ever had an inhibitor?”

COPD, chronic obstructive pulmonary disease.

**Table 2:** Selected patient-centered outcomes evaluated for suitability in hospital comparisons.

Domain	Outcome measure	Variable type	Measurement instrument		
			van Balen *et al*.	Cortesi *et al*.	HiN-6 study
**Clinical outcome**					
Bleeding event	Zero-treated bleeds	Binary	No recommendation	Patient bleeding diaries	Questionnaire item: “Have you had bleeds in the past 12 months?”
	Zero-treated joint bleeds	Binary	No recommendation	Patient bleeding diaries	Questionnaire item: “Have you had joint bleeds in the past 12 months?”
	Annual bleeding rate	Count	No recommendation	Patient bleeding diaries	Questionnaire item: “How many bleeds (joint, mucosal, muscle, wound) have you had in the past 12 months?”
	Annual joint bleeding rate	Count	No recommendation	Patient bleeding diaries	Questionnaire item: “How many joint bleeds have you had in the past 12 months?”
Joint health	Joint score	Continuous	*Joint health was not included*	6MWT	Questionnaire item: “Do you have chronic joint problems (ankle, knee, elbow) due to hemophilia?”
				HJHS	
				HEAD-US score	
Patient-reported outcome					
Physical functioning	General physical functioning	Continuous	PROMIS (physical functioning)	EQ-5D-5L (mobility)	PROMIS-29 (physical functioning)
					SF-36 (physical functioning)
	Physical functioning	Continuous	HAL	HAL	HAL
Social functioning	Social functioning	Continuous	Haemo-Qol-L-A (role functioning)	*Social functioning was not included*	PROMIS-29 (ability to participate in social roles and activities)
			PROMIS (ability to participate in social roles and activities)		SF-36 (social functioning)
Pain	Pain	Continuous	PROBE	BPI v2 Short Form	PROMIS-29 (pain interference)
			PROMIS (pain intencity and interference)		SF-36 (bodily pain)
Mental health	Anxiety	Continuous	Haemo-Qol-L-A (emotional impact)	*Mental health was not included*	PROMIS-29 (anxiety)
			PROMIS (anxiety)		SF-36 (emotional well-being)
	Depression	Continuous	Haemo-Qol-L-A (emotional impact)	*Mental health was not included*	PROMIS-29 (depression)
			PROMIS (depression)		SF-36 (emotional well-being)
Treatment satisfaction	Treatment satisfaction	Continuous	*Treatment satisfaction was not included*	Hemo-SAT	Hemo-SAT

BPI indicates Brief Pain Inventory; EQ-5D-5L, EuroQol 5 Dimensions 5 Levels; Haemo-Qol-L-A, Haemophilia-Specific Quality of Life Questionnaire for Adults; HAL, Haemophilia Activities List; HEAD-US, Haemophilia Early Arthropathy Detection with Ultrasound; Hemo-SAT, Haemophilia Satisfaction; HJHS, Haemophilia Joint Health Score; 6MWT, 6-minute walk test; PROBE, Patient-Reported Outcomes Burdens and Experiences; PROMIS, Patient-Reported Outcomes Measurement Information System; SF-36, 36-Item Short Form Health Survey.

### Statistical analysis

#### Descriptive statistics

Descriptive statistics were computed using the original data before multiple imputation, as the number of missing values was considered low. Data imputation was performed at both the item and patient levels, using item response theory and multiple imputation, respectively (Supplementary Appendix S3). Patient characteristics were presented as counts with percentages or as medians with interquartile ranges (IQRs). Hospital-level outcome scores, defined as numerical values representing the magnitude of outcomes, were calculated according to their variable type: binary outcomes (e.g. zero-treated bleed) were calculated as proportions; count (e.g. annual bleeding rate) and continuous (e.g. physical functioning) outcomes as means. Additionally, IQRs were calculated to quantify the distribution of hospital scores for each outcome.

#### Case-mix effects

The suitability evaluation was conducted using imputed data for both outcomes and case-mix variables in the main analysis. A sensitivity analysis was conducted using complete-case outcome data and imputed case-mix data. All statistical analyses were performed using the R version 4.3.2 (Vienna, Austria) [12].

All case-mix variables were initially included in the analyses; however, given that their relevance may vary across outcomes, backward selection based on the Akaike Information Criterion (AIC) was also performed to identify the most informative predictors for each outcome. The effects of case mix on between-hospital variance were evaluated by first comparing case-mix-adjusted and case-mix-unadjusted hospital-level outcome scores, following the approach described by Vos *et al.* [13]. Adjusted and unadjusted hospital-level outcomes were calculated according to variable type (Supplementary Appendix Table S1). The results of these comparisons were summarized using Spearman’s rank correlation and Lin’s concordance correlation coefficient (CCC) and visualized using scatterplots. These analyses were selected because they are not affected by differences in scale or measurement units, making them suitable for accommodating diverse variable types (binary, count, and continuous) consistent with the heterogeneous outcomes analyzed in this study [14]. They also offer complementary insights: Spearman’s rank correlation examines whether Spearman’s rank correlation examines whether hospital rankings are preserved after case-mix adjustment, whereas Lin’s CCC quantifies how closely the adjusted and unadjusted scores correspond to the 45° line of perfect agreement. Based on conventional thresholds, Spearman’s rank correlation coefficients (*ρ*) were interpreted as very high (0.90–1.00), high (0.70–0.90), moderate (0.50–0.70), low (0.30–0.50), and negligible (0.00–0.30) [15]. Agreement between two scores was interpreted according to McBride: <0.90 poor, 0.90–0.95 moderate, 0.95–0.99 substantial, and >0.99 almost perfect [16].

#### Proportion of between-hospital variation not due to chance (rankability)

The proportion of between-hospital variation not due to chance was examined using rankability [10]. Rankability is calculated as the between-hospital variance from a random-effects model divided by the sum of this variance and the median squared standard error of hospital estimates from a fixed-effects model (Supplementary Appendix Table S2) [17, 18]. Common thresholds applied here for interpreting rankability are low (<0.50), moderate (0.50–0.75), or high (>0.75) [10]. Both the fixed- and random-effects regression models used to assess rankability were adjusted for case mix, with the regression type selected according to the outcome variable (e.g. negative binomial regression for count outcomes).

## Results

### Descriptive statistics

Patients included were adults receiving care at six HTCs, with a median age of 49 years (IQR 33–62), and the majority (84.9%) diagnosed with hemophilia A (Table 3). Substantial heterogeneity in disease severity profiles was observed across HTCs, with the percentage of severe hemophilia ranging from 14.4% to 45.9% (Supplementary Appendix Table S3).

**Table 3:** Description of patient characteristics in the total sample.

Patient characteristics	Total (*N* = 807)	Missing *N* (%)
Hemophilia type, *N* (%)		17 (2.1)
Hemophilia A	685 (84.9)	
Hemophilia B	105 (13.0)	
Age, median [IQR]	49 [33–62]	2 (0.2)
Education, *N* (%)		39 (4.8)
Primary education	45 (5.6)	
Lower secondary	147 (18.2)	
Higher secondary	263 (32.6)	
Bachelor or equivalent	201 (24.9)	
Master or equivalent	112 (13.9)	
Annual income, *N* (%)		183 (22.7)
< €10 000	35 (4.3)	
€10 001–€20 000	66 (8.2)	
€20 001–€30 000	74 (9.2)	
€30 001–€40 000	90 (11.2)	
€40 001–€50 000	69 (8.6)	
€50 001–€60 000	79 (9.8)	
€60 001–€70 000	71 (8.8)	
>€70 000	140 (17.3)	
Severity, *N* (%)		0 (0)
Mild	406 (50.3)	
Moderate	125 (15.5)	
Severe	276 (34.2)	
Inhibitor, *N* (%)		66 (8.2)
Never	657 (81.4)	
Ever	84 (10.4)	
Hypertension, *N* (%)		0 (0)
No	658 (81.5)	
Yes	149 (18.5)	
Hypercholesterolemia, *N* (%)		0 (0)
No	740 (91.7)	
Yes	67 (8.3)	
Diabetes mellitus type 1, *N* (%)		0 (0)
No	794 (98.4)	
Yes	13 (1.6)	
Diabetes mellitus type 2, *N* (%)		0 (0)
No	772 (95.7)	
Yes	35 (4.3)	
COPD, *N* (%)		0 (0)
No	777 (96.3)	
Yes	30 (3.7)	
Psychological complaints, *N* (%)		0 (0)
No	779 (96.5)	
Yes	28 (3.5)	
Hepatitis C, *N* (%)		122 (15.1)
Never	438 (54.3)	
Ever	247 (30.6)	

COPD, chronic obstructive pulmonary disease.

After handling missing values at the item-level using IRT, data completeness exceeded 95% for most outcomes, except for joint score, physical functioning measured with the hemophilia activities list, and treatment satisfaction (Supplementary Appendix Table S4). At the hospital level, for binary outcomes, greater variability was observed in zero-treated bleeds (IQR = 10) compared with zero-treated joint bleeds (IQR = 4) (Table 4). For count outcomes, the IQR of annual bleeding rate (ABR) and annual joint bleeding rate (AJBR) was similar. Continuous outcomes showed minimal variation across hospitals, with IQRs ranging from 0.3 (joint score) to 1.4 (treatment satisfaction).

**Table 4:** Observed hospital-level outcome scores.

Outcome measure	Hospital-level score						IQR
	HTC 1	HTC 2	HTC 3	HTC 4	HTC 5	HTC 6	
**Clinical outcomes**							
Zero-treated bleeds	38.1	44.4	55.3	36.2	50.0	49.3	10.1
Zero-treated joint bleeds	77.1	82.2	78.9	67.9	78.0	82.4	4.0
Annual bleeding rate	2.5	0.9	1.2	2.4	3.3	1.1	1.3
Annual joint bleeding rate	1.51	0.5	0.7	1.2	2.3	0.5	0.9
Joint score	49.2	46.5	48.9	52.1	49.0	49.3	0.3
**Patient-reported outcomes**							
General physical functioning	49.9	50.8	49.5	47.8	49.6	49.2	0.6
Physical functioning (HAL)	51.4	52.5	50.3	48.1	51.0	50.3	1.0
Social functioning	54.7	54.8	52.7	53.5	55.4	54.6	1.0
Pain	48.4	48.5	49.0	50.2	50.0	48.8	1.2
Anxiety	49.0	49.1	49.8	48.0	47.0	48.2	1.0
Depression	47.7	46.7	48.3	46.6	45.5	46.6	0.9
Treatment satisfaction	49.9	51.5	51.4	49.6	48.7	49.7	1.4

HAL, Hemophilia Activities List; HTC, Hemophilia Treatment Center.

### Case-mix effects

In the main analysis, hospital rankings were largely preserved following case-mix adjustment for all clinical outcomes, with very strong correlations observed within the bleeding event domain (*ρ* ≥ 0.83) (Table 5). Case-mix-adjusted hospital scores closely mirror the unadjusted scores, as evidenced by the clustering of points near the diagonal line in the scatterplots (Supplementary Appendix Fig. S2). Unlike clinical outcomes that demonstrated a uniform pattern, the Spearman’s rank correlation coefficients for patient-reported outcomes (PROs) were more variable. Social functioning, anxiety, depression, and treatment satisfaction were less sensitive to case-mix adjustment (*ρ* ≥ 0.77). In contrast, physical functioning and pain showed greater shifts in hospital rankings (*ρ* = 0.09–0.66). Based on Lin’s CCC, nine outcomes demonstrated poor agreement (CCC < 0.90) between the adjusted and the unadjusted hospital scores (Table 5). Moderate agreement was observed only for ABR, AJBR, and treatment satisfaction (CCC 0.93–0.96).

**Table 5:** Case-mix effects and rankability for evaluated outcome measures.

Outcome measure	Case-mix effects		Proportion of between-hospital variation not due to chance
	Consistency Spearman’s CC	Absolute agreement Lin’s CCC	Reliability of differences Rankability
**Clinical outcomes**			
Zero-treated bleeds	0.89	0.86	0.19
Zero-treated joint bleeds	0.89	0.59	0.07
Annual bleeding rate	0.83	0.93	0.55
Annual joint bleeding rate	1.00	0.96	0.58
Joint score	0.77	0.65	0.46
Patient-reported outcomes			
General physical functioning	0.09	0.28	0.08
Physical functioning (HAL)	0.54	0.38	0.12
Social functioning	0.89	0.73	0.02
Pain	0.66	0.63	0.12
Anxiety	0.89	0.79	0.00
Depression	0.94	0.84	0.00
Treatment satisfaction	0.77	0.94	0.32

CC, correlation coefficient; HAL, Hemophilia Activities List.

To minimize the risk of overfitting, we repeated the analyses using AIC-selected case-mix variables and found that the results were similar to those obtained using the full set of case-mix variables (Supplementary Appendix Table S5). Similar findings were obtained in the sensitivity analysis, with results consistent with the main analysis (Supplementary Appendix Table S6).

### Proportion of between-hospital variation not due to chance (rankability)

In the main analysis, the rankability of AJBR was the highest among all evaluated outcomes in this study, at 0.58 (Table 5). Following this, ABR and joint score demonstrated rankability of 0.55 and 0.46, respectively. By contrast, the rankability of zero-treated bleeds and zero-treated joint bleeds was considerably lower, corresponding to 0.19 and 0.07. A similarly low pattern in rankability was observed across nearly all PROs. The highest rankability was only 0.32 for treatment satisfaction, while the remaining PROs ranged from 0.00 to 0.12. The rankability results from the sensitivity analysis were largely consistent with the main analysis (Supplementary Appendix Table S7).

## Discussion

### Statement of principal findings

Interest in applying VBHC to hemophilia care is increasing, and comparing hospitals based on (patient-centered) outcomes is one important strategy for putting these principles into practice. Outcome measures were regarded as suitable to compare hospitals if hospital rankings were largely unaffected by case mix and if a substantial portion of between-hospital variation was not attributable to chance (i.e. high rankability). We found that case-mix adjustment had minimal influence on hospital rankings for most outcomes. Regarding rankability, only ABR and AJBR showed moderate rankability, whereas all other evaluated outcomes had low rankability.

### Strengths and limitations

This study provides a quantitative evaluation of the suitability of patient-centered outcomes for hospital-level comparisons in hemophilia care. Unlike most studies in other fields that mainly focus on clinical outcomes, this study emphasizes patient-centered outcomes developed using VBHC framework and includes both clinical outcomes and PROs measured as binary, count, and continuous variables. Despite these contributions, this study has several limitations. First, we were unable to include all recommended outcomes from the two standardized outcome sets because several outcomes were not available in HiN-6 dataset. While this does not affect the validity of our findings, evaluating all recommended outcomes could have provided a more comprehensive view of their suitability for hospital comparisons in hemophilia care. Second, this analysis included only 807 adults (61.5%) from the HiN-6 study. The resulting small hospital-level samples and low event rates for some outcomes may have limited the statistical power to detect differences between hospitals [17]. Using the complete HiN-6 dataset may modestly increase power and provide a closer reflection of real-world conditions. However, because hemophilia is rare, hospital-level sample sizes would remain relatively small, and our findings likely represent the best achievable estimates given current constraints. Third, the data used in this study were collected in 2019 and therefore do not reflect current treatment strategies in hemophilia care in high-resource settings, such as the use of extended half-life factor concentrates, emicizumab, and gene therapy. However, these treatment patterns may still be representative of practices in low-resource settings, where care continues to rely predominantly on factor replacement therapies. Future studies using more recent data that include newer treatments are warranted to assess whether these factors alter the current findings. Last, the dataset we used included approximately 50% of patients with mild hemophilia, with substantial variation in disease severity between hospitals. The extent to which our findings are generalizable to a subgroup of only severe hemophilia remains uncertain and warrants further investigation. A subgroup analysis was not feasible in this study, as restricting analyses to patients with severe hemophilia would further decrease sample sizes and substantially limit statistical power.

### Interpretation within the context of the wider literature

Nine outcomes evaluated in this study demonstrated high correlations between case-mix-adjusted and case-mix-unadjusted hospital scores, which indicates that case mix had minimal influence on between-hospital variation. Thus, the observed differences between hospitals may reflect true variation in the care being delivered rather than differences in patient characteristics. However, between-hospital variation may persist even after adjusting for case mix due to factors that remain unmeasured or are imperfectly captured, leading to residual confounding [10, 17]. In our analysis, although we incorporated the case-mix variables recommended in the standardized outcome set [6], we could not include all case-mix domains, as well as potential additional variables within each domain (e.g. other comorbidities that may influence outcomes). Incorporating these variables could potentially affect the results by more fully accounting for underlying differences between hospitals. Nevertheless, the presence of residual confounding reflects real-world conditions, as such detailed variables are often not consistently measured or available in routine data collection. Consequently, some degree of unexplained variation is unavoidable and may continue to contribute to observed differences between hospitals.

We also adjusted for case-mix in both fixed- and random-effect regression models used to assess rankability. Rankability reflects the reliability of observed differences between hospitals. Ten evaluated outcomes showed low rankability (<0.50), meaning that more than half of the observed variation between hospitals is due to chance. The low rankability found in most outcomes in this study—particularly the binary outcomes—may be attributed to the large standard errors of the estimated hospital effects from fixed-effect models, primarily driven by the number of patients treated at each hospital (range 76–318). Large standard errors signal low precision, which may contribute to the shrinkage of estimates and the reduction of the between-hospital variance in random-effects models. However, it is important to note that rankability is a group-level measure, indicating whether hospitals can be reliably ranked from best to worst. Low rankability means that the full ranking order should not be interpreted with confidence, but it does not necessarily rule out identifying individual hospitals that perform substantially better or worse than expected.

Although no prior studies have evaluated patient-centered outcomes, including PROs, for hospital-level comparisons in hemophilia, findings from other fields using similar approaches remain informative. For example, Verheul *et al.* conducted a data-driven evaluation of 19 quality indicators (1 structure, 4 outcome, and 14 process indicators) for breast cancer, using registry data from 71 hospitals (*n* = 17 254) [19]. Similar to our findings, this study reported that case mix had limited impact on outcome scores, but all outcomes showed low rankability (<0.50). Likewise, moderate to low rankability was observed in the study by van Dishoeck *et al.*, which evaluated seven outcomes from the Netherlands Inspectorate’s indicator set across 97 hospitals (median sample per hospital 90–277) [17]. Despite the larger sample sizes in these studies, the rankability results were comparable to those observed in our study (*n* = 807), which indicates that attaining high rankability for outcome measures is inherently challenging. It is also important to highlight that the previous studies were conducted using data from quality registries, whereas the present study relied on a nationwide survey not specifically designed for comparing hospitals. This implies that cross-sectional survey data, such as those used in our study, may still provide a feasible approach for evaluating outcomes in a manner comparable to registry-based data. However, rankability improves when outcomes are assessed using longitudinal data collected over multiple years [19, 20]. Therefore, survey data may be less suitable when the goal is to achieve high rankability.

Notably, outcomes with low rankability were predominantly PROs. This finding is important considering the increasing promotion and implementation of PROs in routine care [21, 22]. Previous studies have shown that PROs provide valuable information for individual patient monitoring, shared decision-making, and learning-oriented quality improvement [23]. They also capture meaningful aspects of patients’ health status that are not fully reflected by clinical measures [24–26]. However, our findings suggest that the value of PROs in clinical care does not automatically imply their suitability for hospital-level comparisons, particularly in the context of hemophilia. This was reflected by the superior performance of ABR and AJBR compared with PROs. At the same time, these findings should not be interpreted as a reason to disregard PROs altogether. In the HiN-6 study, PROs were measured cross-sectionally at a single point in the care trajectory. Previous studies in other conditions suggest that when PROs are measured around clinically relevant moments in the patient care trajectory, greater hospital-level variation may be observed [27, 28].

Several factors may explain why ABR and AJBR performed better in terms of rankability than the other evaluated outcomes. First, compared with binary outcomes such as zero treated (joint) bleeds, ABR and AJBR retain more information because they capture the frequency of bleeding events over time rather than reducing bleeding experience to a single threshold [29]. This may allow rate-based measures to better reflect variation between hospitals and provide greater discriminatory ability. Second, ABR and AJBR are direct clinical manifestations of hemophilia and are more closely linked to treatment decisions and clinical management. In contrast, health-related quality of life derived from PROs may be further downstream and may operate partly through clinical outcomes, including bleeding rates and other indicators of disease control. Although reductions in bleeding rates are generally expected to contribute to better quality of life, the extent to which differences in ABR or AJBR translate into measurable differences in PROs remains uncertain. This relationship may be influenced by differences in recall period, timing of assessment, and instrument sensitivity. Third, PROs may capture broader aspects of health beyond bleeding alone, and thus variation in PROs may not be directly attributable to differences in hemophilia care or hospital performance. Together, these factors highlight the need to distinguish outcomes suitable for hospital-level comparison from those valuable for patient-centered care, with ABR and AJBR better suited to the former and PROs remaining essential for the latter.

Although ABR and AJBR demonstrated relatively better performance for hospital comparisons, observed between-hospital differences in these outcomes should still be interpreted with caution. These differences may be influenced by factors beyond our control and may not accurately reflect true between-hospital variation [17]. Such factors may include registration bias, residual confounding, and selection bias [10]. While standardized data collection in the HiN-6 study reduces the likelihood of registration bias, selection bias may still have occurred, potentially influencing which patients were included and affecting the results [30].

### Implications for policy, practice, and research

Undertaking hospital comparisons in the hemophilia field using patient-centered outcomes may be possible, though it requires caution. If such comparisons are pursued, ABR and AJBR are the most appropriate outcomes to use, provided they are interpreted carefully and accompanied by appropriate measures of uncertainty, including funnel plots that account for sample size at each hospital [31–33]. In light of the limited evidence to date, hospital comparisons in this field may be most appropriately carried out in low-stake settings, with the primary aim of enabling mutual learning by exploring between-hospital differences and collaboratively identifying best practices. Furthermore, we found that clinical outcomes generally demonstrate higher reliability compared to PROs. Therefore, future studies should consider evaluating additional clinical outcomes not included in this study, such as the Hemophilia Joint Health Score [9].

## Conclusion

While several consensus-based standardized outcome sets have been developed to support VBHC in hemophilia, their suitability for comparing hospitals rather than individual patients has not been previously evaluated. The present study shows that the evaluated patient-centered outcomes demonstrate low to moderate reliability for hospital-level comparisons, based on the predefined rankability thresholds: low (<0.50), moderate (0.50–0.75), and high (>0.75) [10]. Among these outcomes, clinical outcomes, particularly ABR and AJBR, appeared to be relatively more suitable than PROs for comparing hospitals, as reflected by their moderate rankability. Thus, using ABR and AJBR to compare hospitals may still be useful, as it can promote mutual learning by identifying between-hospital differences and facilitating the collaborative development of best practices in hemophilia care. It is important to note that the lower suitability of PROs for hospital comparisons should not be interpreted as a reason to disregard them altogether. This finding may be specific to the context of hemophilia care and may also be influenced by the use of cross-sectional data collected at a single, nonstandardized point in the care trajectory. Further research is needed to assess whether PROs demonstrate greater reliability for hospital-level comparisons when collected around clinically relevant moments in the patient care trajectory.

## Supplementary Material

mzag105_Supplementary_Data

## Data Availability

The data supporting the findings of this study are not publicly accessible due to ethical and privacy constraints.
