# Supplementary material for: Assessing the real-world value of hemophilia care: how suitable are patient-centered outcomes for hospital-level comparisons?
Source: Int J Qual Health Care. 2026 Aug 4;38(3):mzag105. doi: 10.1093/intqhc/mzag105 (PMC13495108; doi:10.1093/intqhc/mzag105)
Supplement: Supplementary file 1 [file mzag105_supplementary_data.pdf]

Appendix 1

Data

We utilized data from the Hemophilia in the Netherlands-6 (HiN-6) study, a nationwide study conducted in 2018-2019 with the aim of including the entire Dutch hemophilia population.[17] The study was approved by the Medical Ethics Committee of Leiden University Medical Center in 2018, and written informed consent was obtained from all participants. The HiN-6 study involved the collection of data through questionnaires, review of medical records, and blood and urine sampling. Patients received a letter from their physician containing a unique login code to access the electronic questionnaire, with a paper version provided if needed. Clinical data were extracted from medical records using a case report form. A total of 2,192 patients registered at one of the six national hemophilia treatment centers (HTCs) were invited to participate. Of these, 1,312 patients contributed to at least one component of the study, either by completing the questionnaire, consenting to the use of their clinical data, or both. For the present analysis, inclusion criteria required participants to be adults and to have completed both questionnaire data and given consent for data collection from their medical record. Applying these criteria resulted in the inclusion of 807 adult participants from the HiN-6 study.

Appendix 2

Case-mix selection

In previous research, Van Balen et al. specified which case-mix variables should be adjusted for when evaluating outcomes within their core outcome set for hemophilia.[9] We examined the degree to which these case-mix variables could be represented in the HiN-6 dataset by mapping the case-mix domains.[9] Six of the eleven recommended domains were available for inclusion. Notably, Van Balen et al. did not specify which comorbidities should be included as case-mix variables and thus all comorbidity variables available in the HiN-6 dataset were initially considered. Comorbidity variables were tested for correlations with the evaluated outcomes, and those showing statistically significant correlations were selected. Of the case-mix variables recommended by Van Balen et al., availability and access to treatment, degree of joint damage, health literacy, and the specific hemophilia care professionals involved in patient management could not be included because these data were not available in the HiN-6 dataset. Furthermore, sex was excluded as a case-mix variable

because all participants in the HiN-6 study were male. Ultimately, 12 case-mix variables were incorporated.

Outcome measures

We evaluated the extent to which the VBHC core sets could be mapped onto the HiN-6 data to determine which outcomes could be included in the present analysis. This was done by matching the domains of outcomes from the HiN-6 data with the domains from the two VBHC core outcome sets. From van Balen et al., we were able to derive six domains, but four domains could not be obtained from the HiN-6 data (i.e., cure, impact of disease on life expectancy, number of days lost to work or school, and treatment complications).[9] Although data on the number of days lost to work or school were available, the proportion of missing values was too high (>80%), and this domain was therefore excluded. Additionally, treatment-related complications, such as hepatitis C and inhibitor development, were included as comorbidities in the case-mix variables following consultation with a hemophilia expert. From Cortesi et al., five domains were derived, while two domains (i.e., disease activity and osteochondral damage and treatment safety) were not available.[10] Notably, musculoskeletal health was only partially captured through the joint score in the HiN-6 data. Quality of life was measured as health-related quality of life via the Patient-Reported Outcomes Measurement Information System-29 (PROMIS-29) and the Short Form Health Survey-36 (SF-36). Furthermore, although treatment satisfaction is not recognized as an outcome in VBHC, we included it in our analysis because it is part of the outcome set proposed by Cortesi et al.[10] In the end, we were able to evaluate twelve patient-centered outcomes from the HiN-6 data, which included five clinical outcomes and seven patient-reported outcomes.

Appendix 3

Data imputation

For outcomes that are calculated using multiple items, missing values at the item level were handled using the graded response model within item response theory (IRT). Specifically, for physical functioning, social functioning, pain, anxiety, and depression, HiN-6 data were available from both PROMIS-29 and SF-36. Thus, PROMIS-29 scores were derived using concurrent

calibration of responses from both PROMIS-29 and SF-36 items. The IRT approach applied the calibrated PROMIS item parameters alongside the PROsetta Stone parameters for the SF-36 items to align all responses on a common PROMIS metric.[18] We applied this approach to optimize score precision at the individual patient level while simultaneously reducing missing data at item level. A more detailed description of this approach can be found in previous studies.[18-20]

Missing values at patient level in both outcome and case-mix variables were handled using multiple imputation with five imputed datasets and 20 iterations, under the assumption that the data were missing at random. The imputation model incorporated all outcome and case-mix variables, with hospital included as a fixed effect. The imputation method was selected depending on the type of variable, for example using predictive mean matching for continuous variables. No additional variables related to missingness were included in the imputation model beyond the outcomes and case-mix variables. The suitability evaluation was conducted using imputed data for both outcomes and case-mix variables in the main analysis. A sensitivity analysis was conducted using complete-case outcome data and imputed case-mix data.

Table 1 Calculation of adjusted and unadjusted hospital-level scores.

Variable type	Adjusted score	Unadjusted score
Binary variable	Overall score across all hospitals multiplied by the ratio of observed to expected number of events	Event counts divided by the number of patients per hospital
Count variable	Overall score across all hospitals multiplied by the ratio of observed to expected number of events	Event counts divided by the number of patients per hospital
Continuous variable	Sum of the overall mean and the hospital's average residual	Mean scores across all hospitals

Description:

For binary and count outcomes, adjusted hospital-level scores were derived through indirect standardization calculated as the overall score across all hospitals multiplied by the ratio of observed to expected number of events in each hospital. The observed number of events represented the hospital-specific score, whereas the expected number of events was derived from the predicted probability calculated using the case-mix adjustment regression model without hospital identifiers, thereby isolating case-mix effects. For continuous outcomes, adjusted scores were calculated as the sum of the overall mean and the hospital's average residual. Unadjusted (crude) hospital scores were calculated as event counts divided by the number of patients per hospital for binary and count outcomes and as mean scores across all hospitals for continuous outcomes.

Table 2 Formula to calculate rankability.

**Formula**	$$\rho = \frac{\tau^2}{\tau^2 + median({s_i}^2)}$$
**Explanation**	ρ denotes rankability, reflecting proportion of variation that is not due to chance.<br><br>τ^2 denotes the variance component of a random-effects model with hospital specified as a random intercept, reflecting the between-hospital variability.<br><br>s_i the standard error associated with the estimated hospital effect for hospital i, reflecting the precision of the fixed-effect estimates. This measure is largely determined by the number of patients treated at the respective hospital.

Table 3 Patient characteristics for each hemophilia treatment center.

Variable	Hospital case-mix						Mean
	**HTC 1 (M)**	**HTC 2 (L)**	**HTC 3 (L)**	**HTC 4 (H)**	**HTC 5 (L)**	**HTC 6 (M)**	
Hemophilia type (%)							
Hemophilia A	77.1	87.8	73.7	86.8	90.2	87.5	83.86
Hemophilia B	20	10	21.1	11.9	8.54	10.3	13.64
Age (Mean)	45.6	48.3	46.8	47.2	51.3	52	48.53
Education (%)							
Primary education	3.81	8.89	3.95	5.35	4.88	6.62	5.58
Lower secondary	14.3	21.1	19.7	17.6	18.3	19.9	18.48
Higher secondary	29.5	24.4	43.4	36.5	17.1	34.6	30.92
Bachelor or equivalent	25.7	26.7	17.1	25.2	30.5	23.5	24.78
Master or equivalent	18.1	12.2	11.8	11.9	24.4	11	14.92
Annual income (%)							
< €10.000	5.71	0	6.58	5.66	0	4.41	3.73
€10.001 to €20.000	12.4	4.44	10.5	7.23	9.76	7.35	8.62
€20.001 to €30.000	7.62	4.44	19.7	6.60	3.66	16.9	9.83
€30.001 to €40.000	6.67	15.6	14.5	7.86	11	17.6	12.2
€40.001 to €50.000	3.81	8.89	13.2	9.12	9.76	7.35	8.68
€50.001 to €60.000	11.4	15.6	3.95	10.1	8.54	8.09	9.6
€60.001 to €70.000	7.62	8.89	6.58	10.7	8.54	6.62	8.16
>€70.000	18.1	15.6	13.2	17.9	26.8	13.2	17.47
Severity (%)							
Mild	62.9	51.1	47.4	41.5	63.4	54.4	53.45
Moderate	7.62	34.4	18.4	12.6	12.2	16.2	16.91
Severe	29.5	14.4	34.2	45.9	24.2	29.4	29.65
Inhibitor (%)	10.5	11.1	3.95	10.7	15.9	9.56	10.27
Hypertension (%)	16.2	22.2	18.4	17.9	15.9	20.6	18.53
Hypercholesterolemia (%)	7.62	7.78	9.21	9.43	3.66	8.82	7.75
DM type 1 (%)	1.9	3.33	3.95	0.629	1.22	1.47	2.08
DM type 2 (%)	6.67	6.67	3.95	2.83	3.66	5.15	4.82
COPD (%)	5.71	3.33	3.95	3.77	1.22	3.68	3.61
Psychological complaints (%)	3.81	6.67	3.95	3.14	2.44	2.21	3.7
Hepatitis C (%)	28.6	28.9	32.9	33.6	23.2	29.4	29.43

COPD indicates chronic obstructive pulmonary disease; *DM*, diabetes mellitus; *HTC,* hemophilia treatment center.

Patient volume category was defined as follows: L, low-volume center (<100 patients); M, medium-volume center (100–199 patients); H, high-volume center (≥200 patients).

Table 4 Percentage of data available for outcomes.

Domain	Outcome measure	% data available (hospital range)
Bleeding event	Zero-treated bleeds	100 (100-100)
	Zero-treated joint bleeds	99.38 (98.53-100)
	Annual bleeding rate	99.13 (97.56-100)
	Annual joint bleeding rate	99.38 (98.53-100)
Joint health	Joint score	88.72 (83.81-92.22)
Physical functioning	General physical functioning	98.39 (97.06-100)
	Physical functioning (HAL)	89.59 (82.86-95.56)
Social functioning	Social functioning	96.16 (94.12-100)
Pain	Pain	95.42 (93.33-100)
Psychological complaints health	Anxiety	95.91 (94.29-100)
	Depression	95.54 (93.33-100)
Treatment satisfaction	Treatment satisfaction	89.71 (85.37-94.44)

HAL indicates hemophilia activities list.

Table 5 Main results adjusted for case-mix variables selected via backward selection.

Outcome measure	Case-mix effects		Proportion of between-hospital variation not due to chance
	**Consistency** Spearman's CC	**Absolute agreement** Lin's CCC	**Reliability of differences** Rankability
Clinical outcomes			
Zero-treated bleeds	0.89	0.87	0.16
Zero-treated joint bleeds	0.89	0.61	0.10
Annual bleeding rate	0.94	0.92	0.55
Annual joint bleeding rate	0.94	0.91	0.67
Joint score	0.77	0.67	0.48
Patient-reported outcomes			
General physical functioning	0.03	0.25	0.10
Physical functioning (HAL)	0.37	0.37	0.12
Social functioning	0.89	0.72	0.03
Pain	0.43	0.61	0.17
Anxiety	0.94	0.83	0.00
Depression	0.94	0.84	0.00
Treatment satisfaction	0.77	0.95	0.31

CC indicates correlation coefficient; *CCC,* concordance correlation coefficient; *HAL*, hemophilia activities list.

Table 6 Comparisons of Spearman's correlation coefficient and Lin's concordance correlation coefficient between main and sensitivity analyses.

**Outcome measure**	**Main analysis**		**Sensitivity analysis**	
	**Spearman's CC**	**Lin's CCC**	**Spearman's CC**	**Lin's CCC**
Zero-treated bleeds	0.89	0.86	0.89	0.86
Zero-treated joint bleeds	0.89	0.59	0.83	0.00
Annual bleeding rate	0.83	0.93	0.83	0.92
Annual joint bleeding rate	1.00	0.96	1.00	0.96
Joint score	0.77	0.65	0.83	0.71
General physical functioning	0.09	0.28	0.09	0.28
Physical functioning (HAL)	0.54	0.38	0.60	0.50
Social functioning	0.89	0.73	0.71	0.77
Pain	0.66	0.63	0.43	0.61
Anxiety	0.89	0.79	0.94	0.85
Depression	0.94	0.84	1.00	0.88
Treatment satisfaction	0.77	0.94	0.83	0.93

CC indicates correlation coefficient; *CCC,* concordance correlation coefficient; *HAL*, hemophilia activities list.

Table 7 Comparisons of rankability between main and sensitivity analyses.

**Outcome measure**	**Main analysis**			**Sensitivity analysis**		
	τ^2	$median(s_i^2)$	ρ	τ^2	$median(s_i^2)$	ρ
Zero-treated bleeds	0.03	0.11	0.19	0.03	0.18	0.13
Zero-treated joint bleeds	0.01	0.20	0.07	0.02	0.29	0.06
Annual bleeding rate	0.10	0.08	0.55	0.11	0.04	0.71
Annual joint bleeding rate	0.20	0.15	0.58	0.21	0.07	0.75
Joint score	0.69	0.80	0.46	0.79	1.47	0.35
General physical functioning	0.11	1.24	0.08	0.11	1.11	0.09
Physical functioning (HAL)	0.15	1.11	0.12	0.23	0.75	0.23
Social functioning	0.03	1.42	0.02	0.03	0.90	0.03
Pain	0.22	1.61	0.12	0.12	0.91	0.12
Anxiety	0.00	1.49	0.00	0.00	2.24	0.00
Depression	0.00	1.52	0.00	0.00	2.53	0.00
Treatment satisfaction	0.91	1.96	0.32	0.51	1.08	0.32

HAL indicates hemophilia activities list; ρ rankability; τ^2 the variance component of a random-effects model with hospital specified as a random intercept; s_i the standard error associated with the estimated hospital effect for hospital i.

Figure 1 Framework for assessing between-hospital differences in outcome.

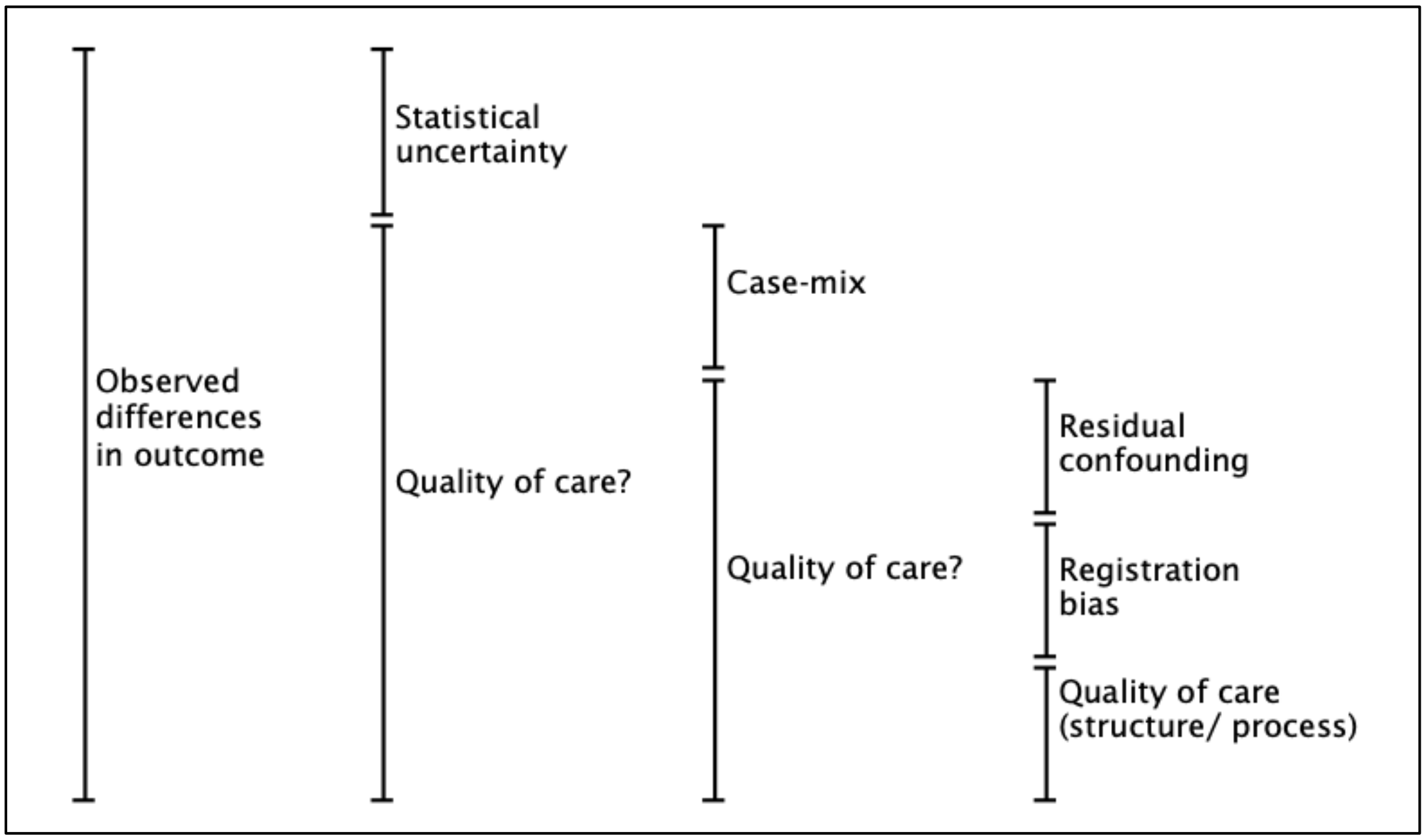

Figure 1 is reproduced with permission from Lingsma HF. *Measuring quality of care: Methods and applications to acute neurological diseases.* Erasmus University Rotterdam; 2010. © 2010 H.F. Lingsma.

Figure 2 Correlation between adjusted and unadjusted hospital level scores.

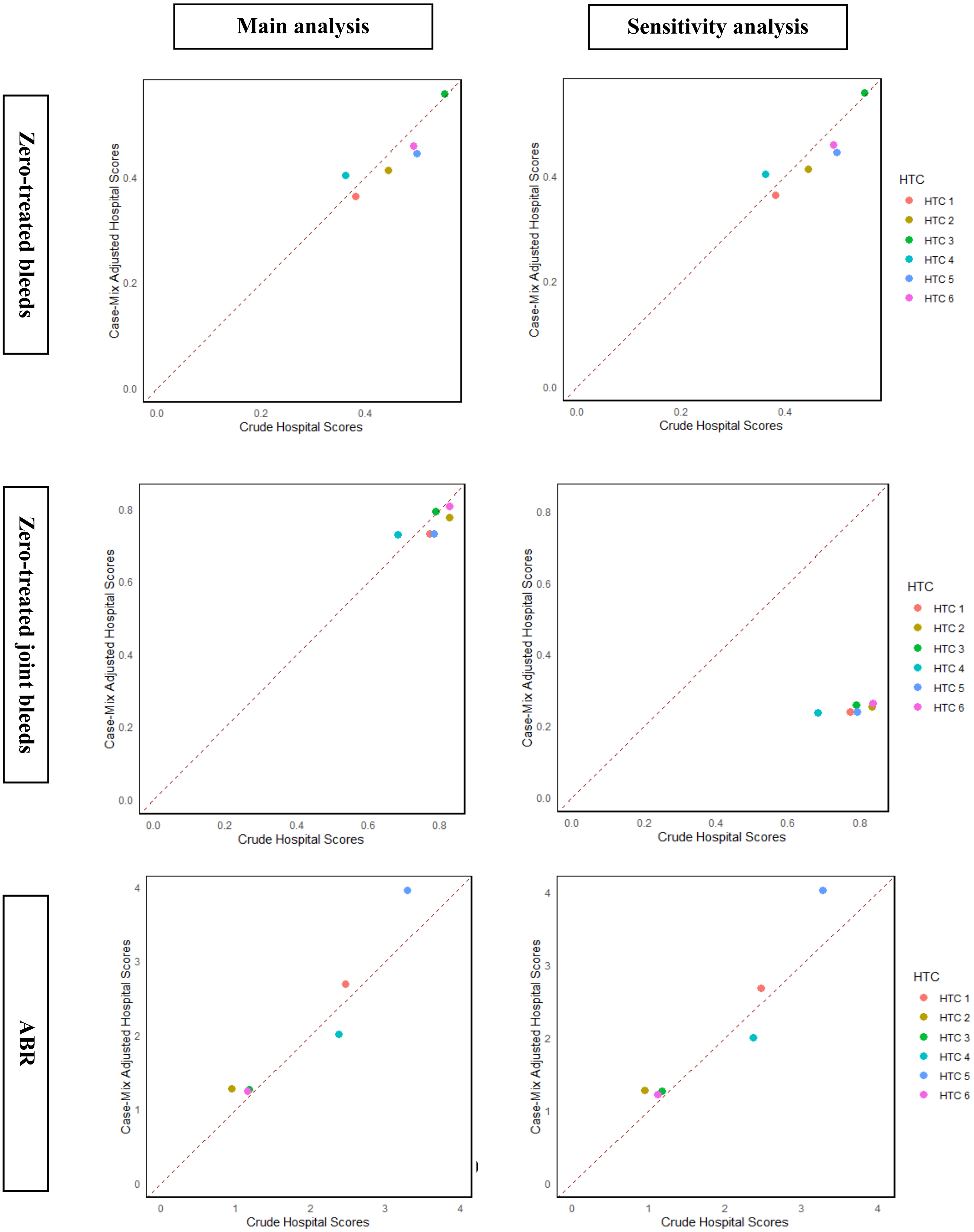

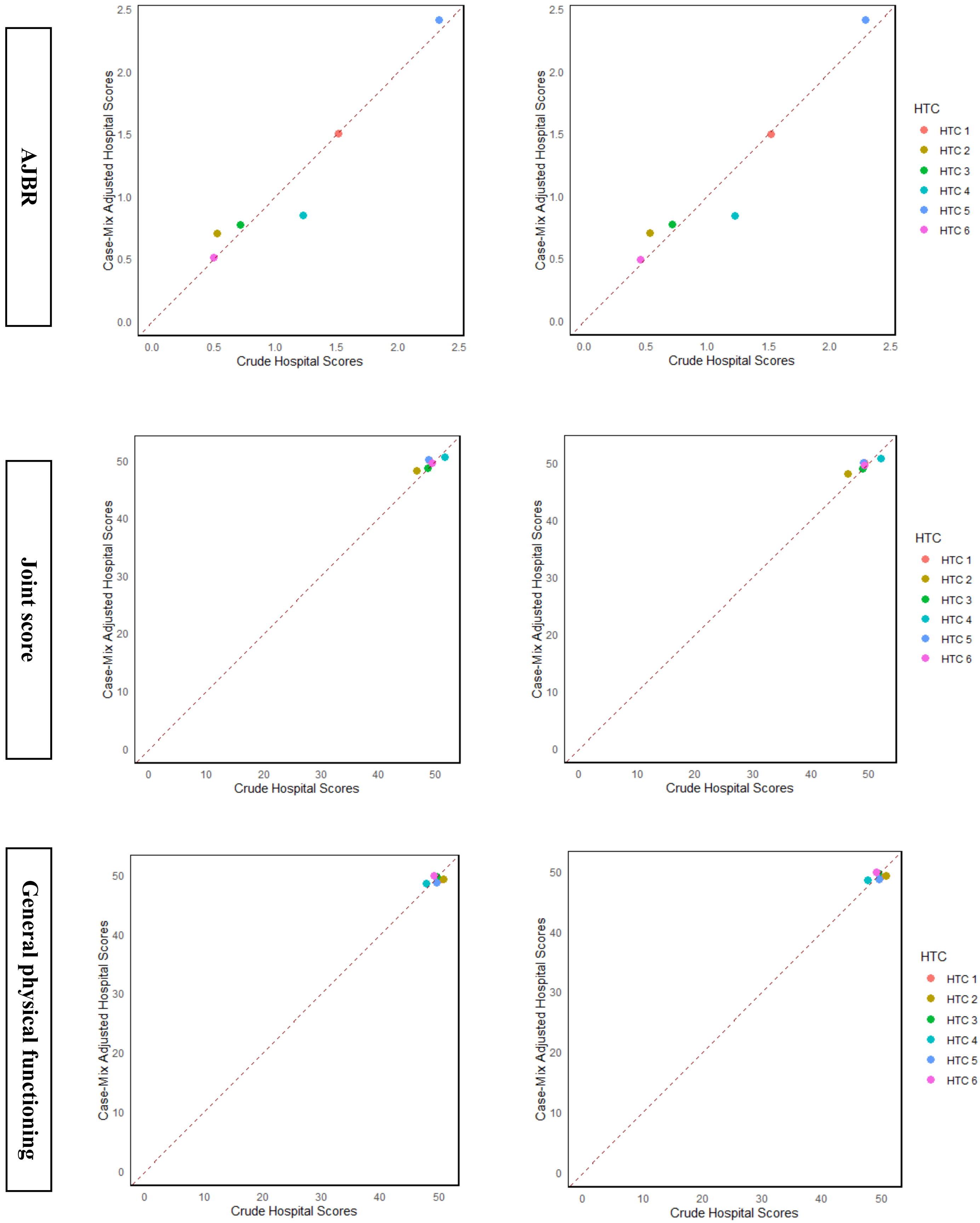

AJBR
Joint score
General physical functioning
Case-Mix Adjusted Hospital Scores
Crude Hospital Scores
HTC
HTC 1
HTC 2
HTC 3
HTC 4
HTC 5
HTC 6

Physical functioning (HAL)

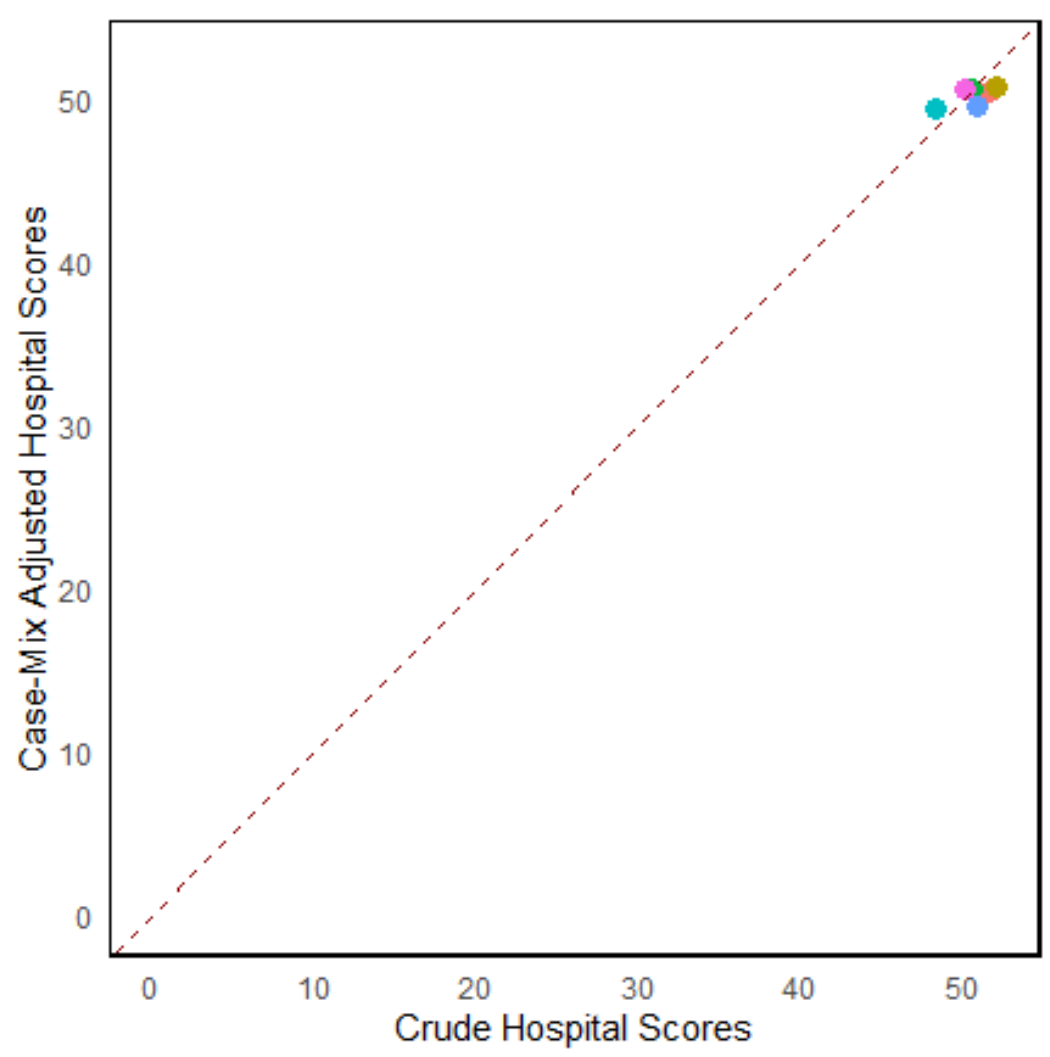

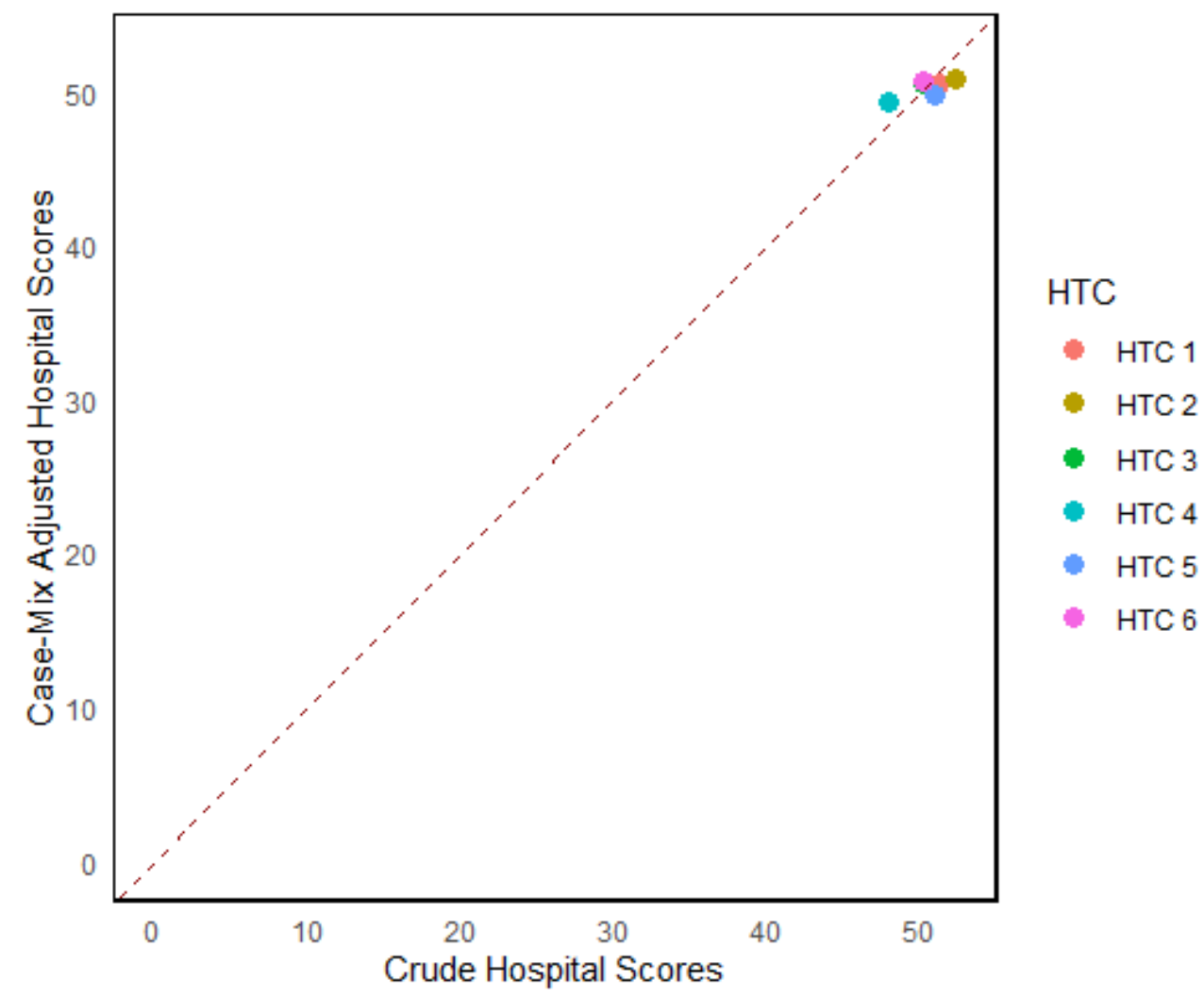

Social functioning

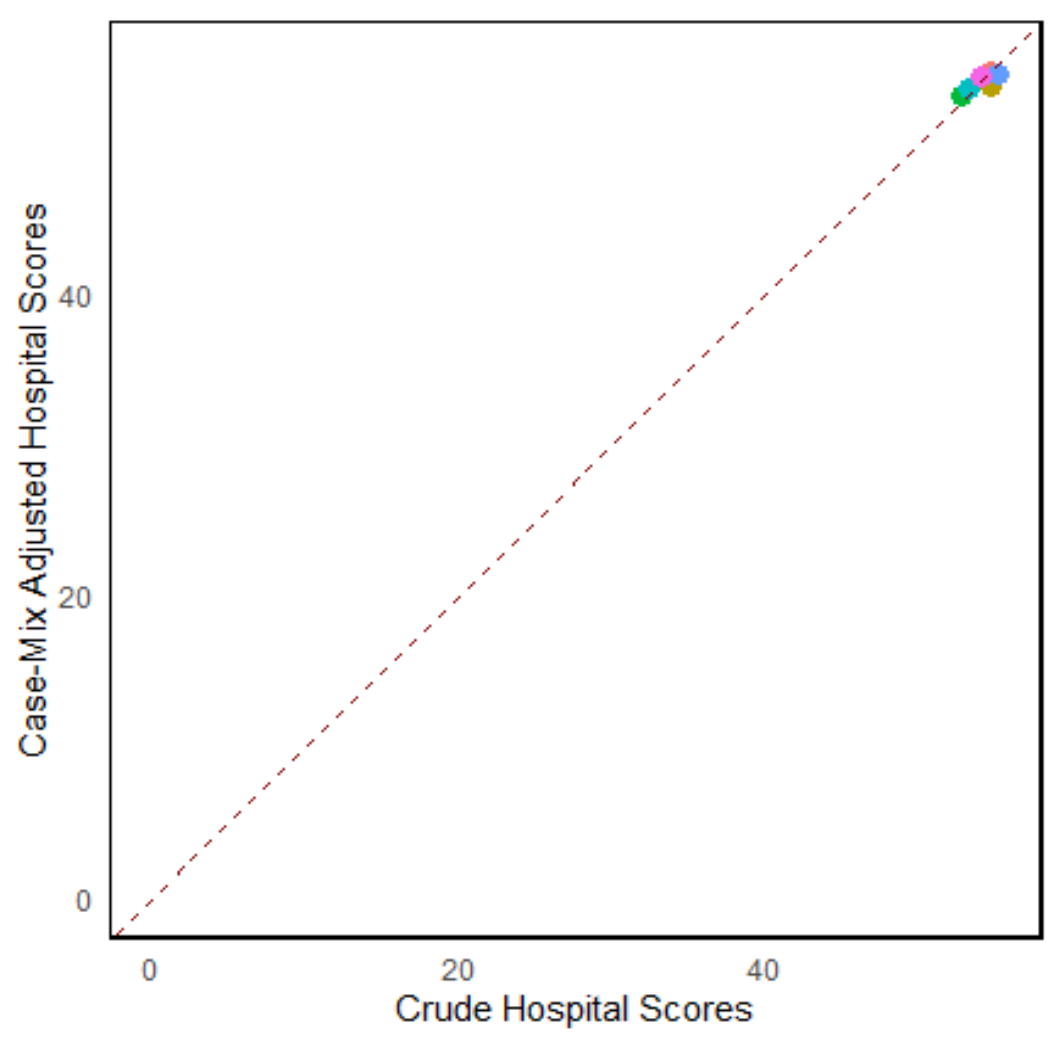

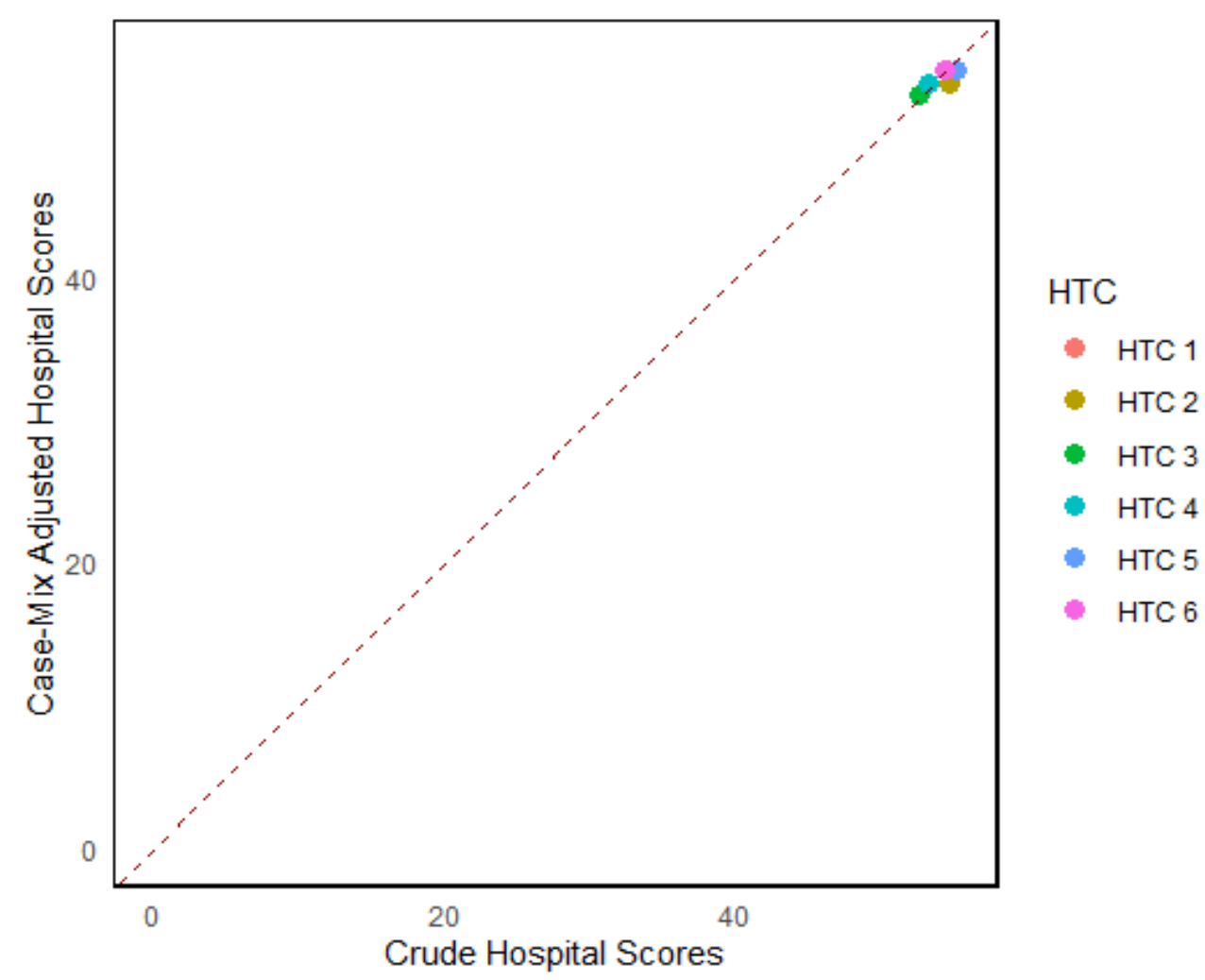

Pain

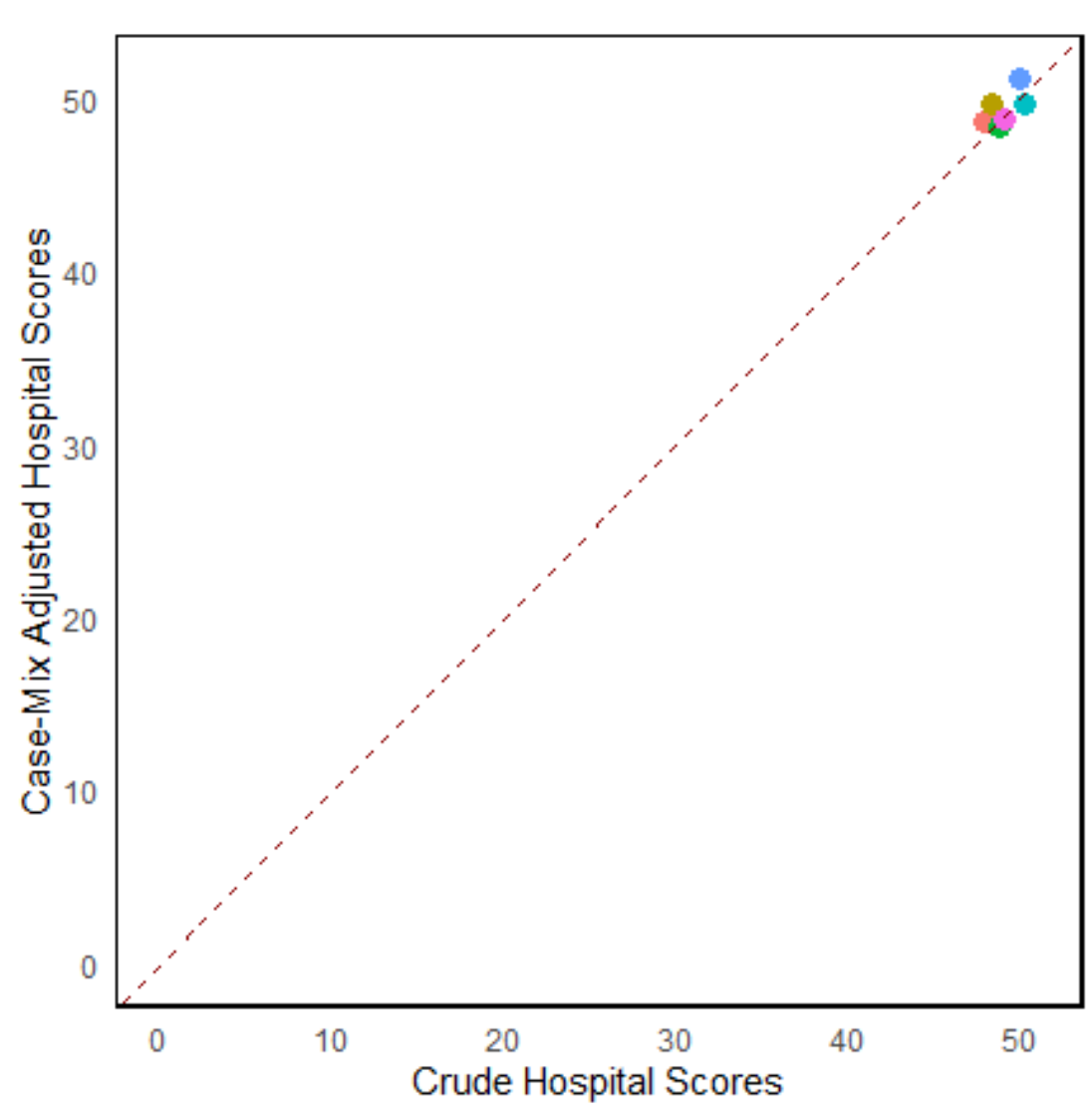

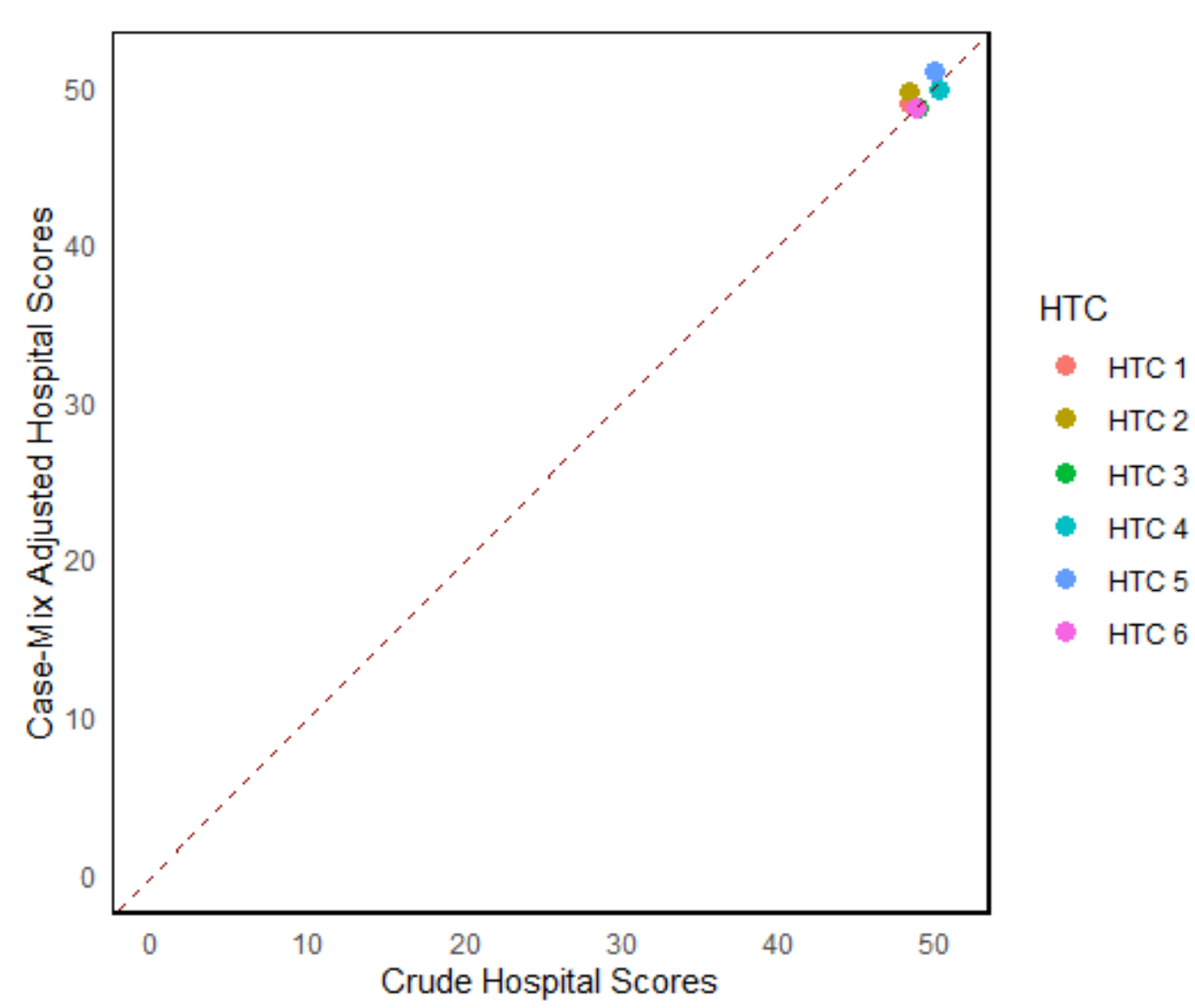

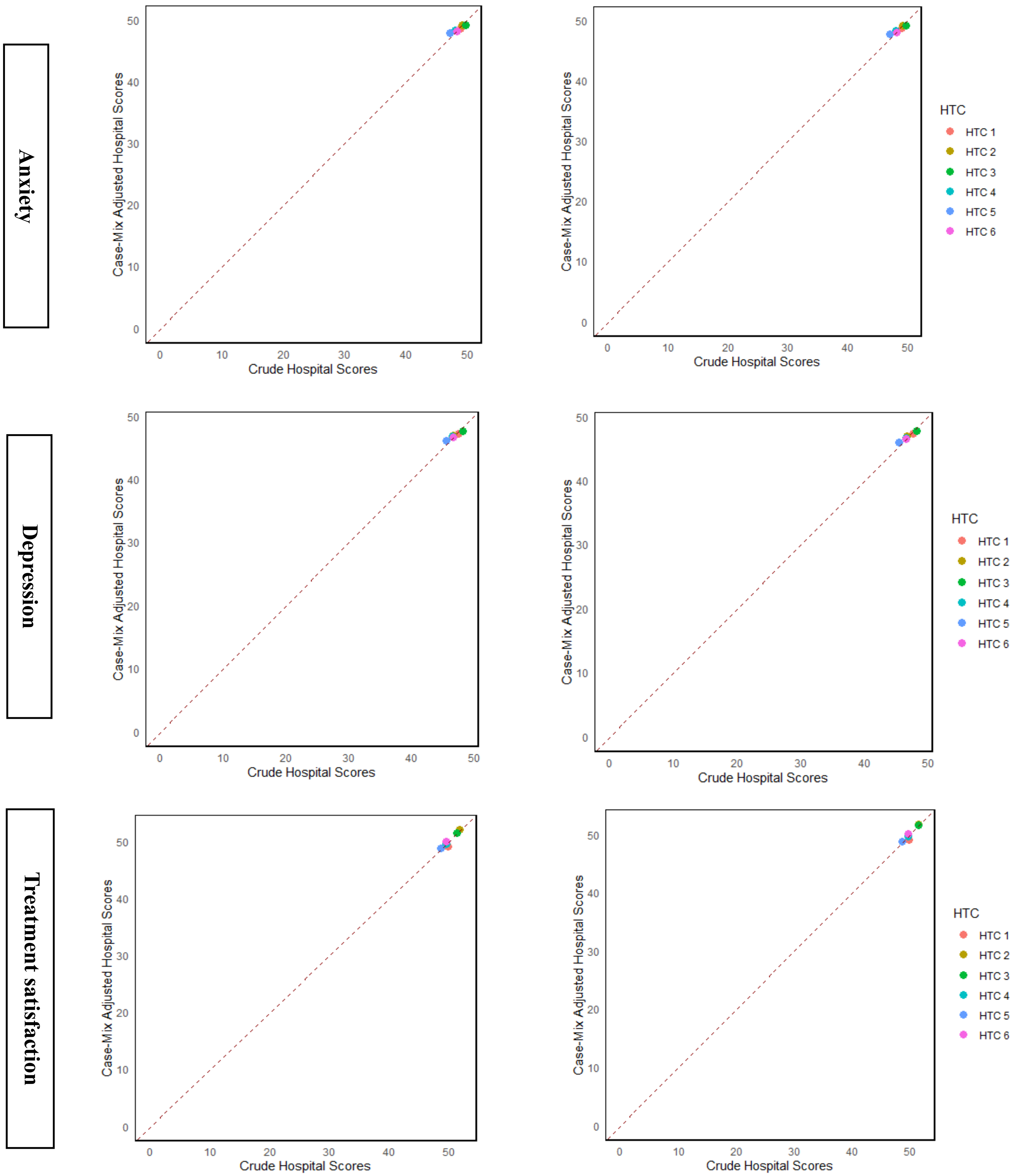

ABR indicates annual bleeding rate; *AJBR,* annual joint bleeding rate; *HAL,* hemophilia activities list; *HTC,* hemophilia treatment center.
